# Analgosedation in Neonatal Intensive Care: Current Strategies, Challenges, and Future Perspectives

**DOI:** 10.3390/life16071185

**Published:** 2026-07-16

**Authors:** Leonardo Detto, Eleonora Alfieri, Anna Munerati, Serafina Perrone, Susanna Esposito

**Affiliations:** 1 Pediatric Clinic, Department of Medicine and Surgery, University Hospital of Parma, 43126 Parma, Italy; leonardo.detto@unipr.it (L.D.); ele.alfieri95@gmail.com (E.A.); anna.munerati@unipr.it (A.M.); 2 Neonatology Unit, Department of Medicine and Surgery, University Hospital of Parma, 43126 Parma, Italy; serafina.perrone@unipr.it

**Keywords:** neonatal intensive care, analgosedation, neonatal pain, procedural analgesia, non-pharmacological interventions, pain assessment

## Abstract

Pain and stress are frequent and clinically relevant challenges in neonatal intensive care, particularly among preterm and critically ill newborns exposed to repeated invasive procedures, mechanical ventilation, surgery, and advanced life-support interventions. Effective analgosedation is essential to reduce discomfort, attenuate physiological instability, improve tolerance of intensive care procedures, and potentially limit adverse neurodevelopmental consequences. However, neonatal pain and analgosedation management remain complex because of developmental immaturity, pharmacokinetic and pharmacodynamic variability, and the need to balance adequate analgosedation against treatment-related complications. This narrative review summarizes current evidence on analgosedation in the Neonatal Intensive Care Unit, focusing on clinical indications, pharmacological agents, non-pharmacological strategies, monitoring tools, adverse effects, and future perspectives. Opioids, benzodiazepines, dexmedetomidine, and ketamine each have specific potential benefits and limitations, requiring individualized selection, careful titration, and continuous reassessment. Non-pharmacological interventions, including oral sucrose, non-nutritive sucking, facilitated tucking, breastfeeding, skin-to-skin care, and environmental modulation, should be integrated into multimodal pain-management protocols. Validated instruments such as COMFORTneo, N-PASS, and PIPP-R support standardized assessment and guide therapeutic decisions. Future advances may derive from objective monitoring technologies, artificial intelligence, developmental pharmacology, and precision-medicine approaches. A multidisciplinary, protocol-driven, and family-centered strategy is essential to optimize neonatal comfort while minimizing avoidable drug exposure.

## 1. Introduction

Pain exposure is an unavoidable component of neonatal intensive care, particularly among preterm and critically ill newborns who often require prolonged hospitalization and repeated invasive procedures. Endotracheal intubation, mechanical ventilation, vascular catheterization, postoperative care, airway suctioning, and repeated blood sampling are among the many diagnostic and therapeutic interventions routinely performed in the Neonatal Intensive Care Unit (NICU). Although these procedures are frequently lifesaving, recurrent nociceptive stimulation during early life may adversely affect neurological, physiological, and behavioral development when pain is not adequately recognized and treated [1]. Accordingly, analgosedation has become a central component of neonatal critical care, with goals extending beyond immediate comfort to include physiological stabilization, stress reduction, and potential neuroprotection.

The neonatal nervous system has distinctive developmental characteristics that increase vulnerability to painful stimuli. By 24–28 weeks of gestation, ascending nociceptive pathways are sufficiently developed to transmit painful input to higher cortical structures, whereas descending inhibitory pathways involved in pain modulation remain immature [2]. This imbalance may amplify pain responses and prolong their physiological consequences, particularly in extremely preterm infants. Neuroimaging studies using functional magnetic resonance imaging and near-infrared spectroscopy have demonstrated cortical activation after noxious stimulation even in very premature neonates, supporting the presence of central pain processing during early developmental stages [3]. Repeated or untreated pain has been associated with altered cerebral maturation, dysregulated hypothalamic–pituitary–adrenal axis activity, modified somatosensory processing, and adverse cognitive, behavioral, and pain-related outcomes later in childhood [4].

Despite increasing recognition of neonatal pain and its potential long-term consequences, analgosedation in the NICU remains clinically complex. Neonates, especially those born preterm, show marked variability in drug metabolism, receptor sensitivity, organ maturation, body composition, and physiological reserve. These factors profoundly influence the pharmacokinetic and pharmacodynamic profiles of agents for analgosedation, increasing the risk of respiratory depression, hypotension, gastrointestinal dysmotility, tolerance, withdrawal syndrome, and possible interference with normal brain development. Conversely, inadequate analgosedation may exacerbate stress responses, impair ventilator synchrony, destabilize cardiorespiratory function, and contribute to cumulative neurodevelopmental injury. Therefore, neonatal analgosedation requires a careful balance between effective pain control, adequate sedation, and patient safety.

Current neonatal care increasingly promotes individualized, protocol-driven, and multimodal strategies that integrate pharmacological treatment with non-pharmacological supportive interventions [1]. Such approaches should be guided by validated pain, agitation, and analgosedation assessment tools and adapted to gestational age, postnatal age, clinical condition, type of procedure, and expected duration of exposure. Continuous reassessment, cautious dose titration, and multidisciplinary collaboration are essential to optimize comfort while limiting treatment-related complications.

This narrative review critically examines current evidence on analgosedation practices in neonatal intensive care. Particular attention is given to the neurobiological basis of neonatal pain, clinical indications for analgosedation, pharmacological characteristics of commonly used agents, non-pharmacological interventions, validated assessment instruments, and major complications associated with analgosedation exposure. Finally, emerging perspectives for safer, more objective, and individualized neonatal pain management strategies are discussed.

## 2. Methods

A comprehensive literature search was conducted to identify relevant evidence on analgosedation practices in neonates admitted to NICUs. The search was performed in PubMed/MEDLINE and the Cochrane Library. The literature search covered publications from 1 January 2000 to 31 March 2026 in order to include contemporary evidence reflecting current neonatal intensive care practice. Older landmark studies were also considered when they provided foundational information on neonatal pain physiology, developmental neurobiology, or historically important clinical evidence.

The search strategy combined Medical Subject Headings (MeSH) and free-text terms related to neonatal pain, analgosedation, and intensive care. The principal search terms included: “neonatal intensive care”, “newborn”, “preterm infant”, “premature neonate”, “analgosedation”, “sedation”, “analgesia”, “pain management”, “procedural pain”, “mechanical ventilation”, “endotracheal intubation”, “opioids”, “morphine”, “fentanyl”, “benzodiazepines”, “midazolam”, “dexmedetomidine”, “ketamine”, “non-opioid analgesics”, “sucrose”, “skin-to-skin care”, “kangaroo care”, “breastfeeding”, “facilitated tucking”, and “non-pharmacological interventions”. Boolean operators such as “AND” and “OR” were used to combine search terms and refine the results according to topic relevance.

Eligible publications included randomized and non-randomized clinical trials, observational studies, systematic reviews, meta-analyses, narrative reviews, pharmacological studies, expert consensus documents, and clinical practice guidelines. Priority was given to articles addressing pharmacological and non-pharmacological approaches to pain control, analgosedation, and comfort management in neonates, particularly preterm or critically ill infants requiring intensive care. Studies focusing on commonly used agents for analgosedation, including opioids, benzodiazepines, alpha-2 agonists, N-methyl-D-aspartate (NMDA) receptor antagonists, and non-opioid analgesics, were considered relevant. Articles evaluating validated neonatal pain and analgosedation assessment tools, adverse effects of analgosedation exposure, withdrawal, tolerance, and long-term neurodevelopmental outcomes were also included.

Only articles published in English were considered. Studies were excluded if they were not directly related to neonatal populations, did not address analgosedation or pain assessment, or focused exclusively on pediatric or adult intensive care without neonatal-specific data.

The selection process was based on relevance to the objectives of this narrative review. Titles and abstracts were screened to identify potentially eligible articles, and full texts were reviewed when the content was considered pertinent to neonatal analgosedation. Additional references were identified by manually reviewing the bibliographies of selected articles, guidelines, and review papers. Particular attention was given to evidence addressing the balance between effective analgosedation, patient safety, and potential neurodevelopmental consequences.

Given the narrative nature of this review, no formal risk-of-bias assessment or quantitative data synthesis was performed. Instead, the available literature was critically analyzed and organized thematically into major areas of interest, including the neurobiological basis of neonatal pain, clinical indications for analgosedation, pharmacological agents, non-pharmacological strategies, pain and analgosedation assessment tools, monitoring for adverse effects, and future perspectives. This approach was intended to provide a broad, clinically oriented overview of current evidence and remaining uncertainties in the management of pain and analgosedation in neonatal intensive care.

## 3. Indications for Analgosedation in the Neonatal Intensive Care Unit (NICU)

Analgosedation is an essential component of neonatal intensive care, with both therapeutic and supportive functions in critically ill newborns exposed to painful, stressful, or prolonged invasive interventions. Its use is not limited to the relief of acute pain or distress; rather, it aims to reduce nociceptive-induced physiological instability, attenuate metabolic and hormonal stress responses, improve cardiorespiratory adaptation, and potentially limit the long-term consequences of repeated untreated pain during a vulnerable period of neurodevelopment [5,6]. In this context, the decision to initiate analgosedation should be individualized according to gestational age, clinical condition, procedure type, anticipated duration of exposure, and the balance between expected benefits and possible adverse effects.

One of the most frequent indications for analgosedation in the NICU is invasive respiratory support, particularly endotracheal intubation and mechanical ventilation [7]. This is especially relevant in preterm infants with respiratory distress syndrome, evolving bronchopulmonary dysplasia, pulmonary hypertension, postoperative respiratory failure, or severe cardiorespiratory instability. Mechanical ventilation may cause significant discomfort through endotracheal tube irritation, ventilator–patient asynchrony, repeated airway suctioning, restricted movement, and prolonged exposure to environmental stressors. If inadequately controlled, pain and agitation may increase oxygen consumption, catecholamine release, pulmonary vascular resistance, and metabolic demand, while also impairing ventilatory synchrony and contributing to hemodynamic instability. Appropriately titrated analgosedation may improve tolerance of mechanical ventilation, promote patient–ventilator synchrony, reduce stress responses, and facilitate safer delivery of respiratory support [7].

Although available clinical guidelines differ in scope and level of detail, several consistent recommendations can be identified for mechanically ventilated neonates, procedural analgosedation, and intubation premedication. For mechanically ventilated neonates, routine continuous opioid or sedative administration should not be used solely because an infant is intubated. Instead, treatment should be individualized according to pain scores, agitation, ventilator synchrony, underlying disease, postoperative status, and expected duration of respiratory support. Evidence from Cochrane reviews does not support routine opioid administration for all ventilated neonates, because morphine or fentanyl probably has little or no effect on duration of mechanical ventilation or mortality, and evidence regarding pain and long-term neurodevelopmental outcomes remains uncertain [7]. When analgosedation is clinically indicated, opioids such as morphine or fentanyl remain commonly used agents, but they should be titrated to the lowest effective dose, reassessed frequently, and accompanied by monitoring for respiratory depression, hypotension, feeding intolerance, tolerance, and withdrawal. Non-pharmacological comfort measures, including positioning, facilitated tucking, clustering of care, environmental control, and parental involvement, should be maintained whenever feasible to reduce stress and cumulative drug exposure [8]. In neonates receiving prolonged infusions, daily assessment of ongoing need and structured weaning plans are recommended to minimize tolerance and iatrogenic withdrawal.

For procedural analgosedation, guideline-based care recommends matching the analgosedation strategy to the expected intensity and duration of pain [9]. For minor procedures, including heel lance, venipuncture, peripheral intravenous cannulation, and routine blood sampling, first-line measures include oral sucrose or glucose, non-nutritive sucking, breastfeeding when feasible, skin-to-skin care, facilitated tucking, swaddling, and reduction of environmental stressors [6]. Recent Canadian Paediatric Society guidance emphasizes that institutions should implement a multidimensional newborn pain-management framework that includes staff education, minimization of painful procedures, structured assessment and reassessment, pharmacological and non-pharmacological interventions, and active parental engagement in pain care [6]. For moderately or severely painful procedures, such as chest tube insertion or removal, lumbar puncture, central vascular access, postoperative wound manipulation, or other invasive bedside interventions, non-pharmacological measures should be combined with local anesthesia and, when indicated, systemic analgosedation such as fentanyl, morphine, or acetaminophen according to the infant’s clinical condition and local protocols. Topical or infiltrative local anesthetics may be useful for selected procedures, but clinicians should consider gestational age, skin maturity, dosing limits, and potential toxicity.

For endotracheal intubation and laryngoscopy, premedication is recommended for non-emergent procedures unless immediate resuscitation or life-threatening instability precludes it [10]. The American Academy of Pediatrics clinical report states that neonatal intubation is painful and physiologically destabilizing and that premedication improves intubating conditions, decreases procedure duration and number of attempts, and reduces airway trauma and adverse physiological responses [10]. More recent Canadian Paediatric Society guidance similarly recommends that non-emergent neonatal laryngoscopy/intubation include a vagolytic agent, a fast-acting opioid, and, when endotracheal intubation is performed, a short-acting neuromuscular blocker, with continuous cardiorespiratory monitoring and performance or supervision by an experienced provider [11]. A commonly cited regimen includes atropine 0.01–0.02 mg/kg, fentanyl 2 mcg/kg administered slowly over 2–5 min to reduce the risk of chest wall rigidity, and succinylcholine 2 mg/kg when neuromuscular blockade is appropriate [11]. For less invasive surfactant administration techniques requiring laryngoscopy, non-pharmacological measures should be prioritized, and fast-acting opioid analgosedation with a vagolytic may be considered according to the infant’s clinical stability, procedural technique, and local expertise [11]. Emergency intubation during active resuscitation remains an exception in which premedication may not be feasible.

Overall, these recommendations support a practical approach in which NICU analgosedation is tailored to the clinical context rather than applied routinely. The guiding principles are to prevent avoidable pain, use validated assessment tools, combine non-pharmacological and pharmacological strategies, premedicate for non-emergent airway procedures, avoid unnecessary prolonged infusions, and implement structured monitoring and weaning protocols.

Therapeutic hypothermia for hypoxic–ischemic encephalopathy represents a particularly complex indication for analgosedation [12]. During cooling, neonates may experience discomfort related to thermal stress, invasive monitoring, mechanical ventilation, and underlying encephalopathy. At the same time, hypothermia modifies drug pharmacokinetics and pharmacodynamics by reducing hepatic metabolism, renal clearance, and tissue distribution, thereby increasing the risk of drug accumulation and prolonged sedative effects. Analgosedation regimens in this setting should therefore be carefully individualized, with close monitoring for respiratory depression, hypotension, delayed clearance, and excessive sedation [12].

Analgosedation may also be necessary in neonates receiving advanced life-support modalities, including extracorporeal membrane oxygenation, high-frequency oscillatory ventilation, high-frequency jet ventilation, inhaled nitric oxide combined with invasive ventilation, or other forms of complex cardiorespiratory support [13]. In these situations, adequate comfort, immobility, and tolerance of invasive devices may be essential to prevent accidental extubation, cannula displacement, ventilator dyssynchrony, and clinical deterioration. However, these infants are often exposed to prolonged analgosedation infusions, increasing the risk of tolerance, withdrawal syndrome, gastrointestinal dysmotility, hemodynamic instability, and potential neurodevelopmental concerns. The clinical challenge is therefore to achieve sufficient analgosedation while minimizing cumulative drug exposure through regular reassessment and structured weaning strategies [13].

Finally, analgosedation should be considered in neonates with severe agitation, refractory distress, or pain related to specific disease states, such as necrotizing enterocolitis, severe skin injury, fractures, palliative care conditions, or invasive bedside interventions [13]. In all cases, treatment should be guided by validated pain and analgosedation assessment tools and embedded within a multimodal strategy that includes environmental control, developmental care, parental involvement, and non-pharmacological analgosedation measures. The overarching goal is to provide effective pain relief and comfort while preserving physiological stability, supporting neurodevelopment, and reducing avoidable exposure to analgosedation medications.

## 4. Pharmacological Agents

Table 1 summarizes the main pharmacological agents used for analgosedation in the NICU. Drug selection should always be individualized according to gestational age, postnatal age, weight, clinical condition, organ maturity, hepatic and renal function, hemodynamic status, respiratory support, and expected duration of treatment. In neonates, and particularly in extremely preterm infants, pharmacokinetic and pharmacodynamic variability is substantial; therefore, careful dose titration, repeated pain and analgosedation assessment, and close monitoring for adverse effects are essential.

### 4.1. Opioids

Opioids remain among the most frequently used drugs for neonatal analgosedation, particularly during invasive mechanical ventilation, postoperative care, and painful procedures. Morphine and fentanyl are the most commonly used agents, although their pharmacological profiles differ substantially. Morphine has a slower onset of action and may be associated with histamine release, hypotension, delayed gastrointestinal motility, and prolonged clearance in preterm neonates. Fentanyl, by contrast, is a highly lipophilic synthetic opioid characterized by rapid onset, short duration of action after single doses, and relatively limited hemodynamic effects when administered appropriately [14].

Fentanyl is commonly used to provide rapid analgosedation in controlled clinical settings, particularly before painful procedures or during mechanical ventilation. Because of its potency and rapid onset, administration should occur under continuous cardiorespiratory monitoring, with staff prepared to recognize and manage potential adverse effects, including bradycardia, laryngospasm, respiratory depression, and chest wall rigidity, especially after rapid bolus administration [14,15,16,17]. Compared with morphine, fentanyl is often preferred in hemodynamically unstable preterm infants because it is less frequently associated with hypotension and has a more rapid analgesic effect.

European and Italian clinical experience suggests that fentanyl is frequently preferred over morphine in NICU practice because of its faster onset of action, shorter duration after intermittent administration, and lower risk of hypotension, particularly in preterm infants [18]. A single-center randomized controlled trial reported that fentanyl infusion at 1.5 mcg/kg/h provided analgesia without significant adverse respiratory effects or difficulty in weaning from mechanical ventilation [18]. However, compared with morphine, fentanyl may be associated with a higher risk of tolerance and withdrawal, particularly after prolonged continuous infusion [19]. For this reason, intermittent fentanyl boluses may be preferable for short painful procedures, especially in extremely preterm neonates, infants younger than 27 weeks’ gestation, or newborns at increased risk of hypotension [20].

Clinical practice varies considerably among NICUs. Some centers use continuous opioid infusions, while others rely primarily on intermittent boluses, and many combine both strategies. European data indicate a frequent tendency to combine continuous infusion with bolus dosing, whereas bolus-only regimens are less commonly used [19]. Nevertheless, intermittent opioid boluses may be particularly useful for procedural pain and for selected ventilated infants, as they may reduce cumulative opioid exposure while maintaining adequate analgesic efficacy [19]. This approach may be especially relevant in less severely ill but very preterm infants, in whom unnecessary prolonged opioid exposure should be avoided.

The use of strong analgosedation in neonates requires particular caution because of concerns regarding potential neurodevelopmental effects. Preclinical studies have suggested that opioids may interfere with brain maturation by affecting neuronal differentiation, proliferation, apoptosis, and synaptic development [19]. At the same time, repeated untreated pain is itself associated with adverse neurodevelopmental outcomes, including altered myelination, impaired cognitive performance, and changes in pain sensitivity later in childhood [20]. Therefore, the clinical objective is not to avoid analgosedation, but to provide adequate pain relief while minimizing unnecessary or prolonged drug exposure.

Available evidence supports the use of opioids when clinically indicated. Opioid analgosedation during mechanical ventilation can reduce pain and stress responses without consistently prolonging ventilation or increasing major short-term adverse outcomes. However, current evidence does not support routine opioid administration for all mechanically ventilated neonates irrespective of pain scores or clinical need [7]. Opioid treatment should therefore be guided by validated pain and analgosedation assessment tools, individualized according to the infant’s condition, and reassessed frequently.

Premedication with analgosedation agents is recommended before elective or semi-elective endotracheal intubation. A commonly used regimen includes atropine, fentanyl administered slowly, and a neuromuscular blocking agent such as succinylcholine or rocuronium when clinically appropriate [21,22,23]. Fentanyl should be administered over several minutes to reduce the risk of chest wall rigidity. In selected cases, such as extremely low birth weight infants, severely asphyxiated neonates, or infants with suspected neuromuscular disease, muscle relaxants may be avoided or used with particular caution [18].

Treatment duration is a key determinant of opioid-related complications. Short-acting opioids are generally preferred for procedural pain, whereas longer-acting agents may be more appropriate for sustained postoperative or disease-related pain. A strategy based on an initial dose sufficient to achieve rapid analgesia, followed by dose reduction according to pain scores, may help limit cumulative exposure and reduce the development of tolerance. Escalation of opioid dose should be considered only after careful reassessment of pain, ventilator synchrony, disease progression, and alternative causes of agitation. Tolerance may become clinically relevant after several days of fentanyl infusion and after longer courses of morphine [1,18].

Structured weaning protocols are important to prevent and recognize iatrogenic withdrawal syndrome. When opioid therapy has lasted fewer than five days, the dose may often be reduced more rapidly, for example by 30–50% initially, followed by further stepwise reductions if tolerated. After longer treatment courses, particularly with continuous infusions, a slower taper is advisable, such as reducing the dose by approximately 20% during the first 24 h and then by smaller decrements thereafter [18]. During weaning, neonates should be evaluated regularly for withdrawal symptoms, including tremors, irritability, high-pitched crying, feeding intolerance, sleep disturbance, autonomic instability, and seizures. Structured assessment tools, including modified neonatal withdrawal scores such as the Finnegan score, may support early recognition and guide treatment adjustments [24].

### 4.2. Benzodiazepines

Midazolam is a short-acting benzodiazepine used primarily for sedation rather than analgesia. It acts by binding to the gamma-aminobutyric acid type A receptor and enhancing the inhibitory effect of GABA, thereby increasing chloride channel opening, neuronal hyperpolarization, and reducing neuronal excitability [25]. Clinically, this results in sedation, anxiolysis, anticonvulsant activity, muscle relaxation, and anterograde amnesia. Its rapid onset and water solubility have contributed to its use for procedural and continuous sedation in neonatal intensive care.

However, the pharmacokinetics of midazolam in neonates differ markedly from those observed in older children and adults. Midazolam is metabolized primarily in the liver by cytochrome P450 3A4 and 3A5 enzymes to the active metabolite 1-hydroxymidazolam, which is subsequently glucuronidated and eliminated by the kidneys [25]. In preterm infants, immature hepatic metabolism and reduced renal clearance can lead to drug accumulation and prolonged sedation. The elimination half-life may be markedly prolonged in extremely preterm neonates, reaching 22–30 h compared with approximately 2–6 h in adults [25].

From a pharmacodynamic perspective, midazolam may cause dose-dependent respiratory depression and cardiovascular instability [2,16]. Hypotension may result from systemic vasodilation and negative inotropic effects, and the risk can be amplified when midazolam is administered together with opioids. Hypotension is particularly concerning in preterm infants because it may reduce cerebral perfusion and oxygen delivery during a period of high vulnerability [2,16]. Respiratory depression, delayed extubation, paradoxical agitation, myoclonus, and prolonged sedation are additional concerns.

Potential neurodevelopmental effects represent another important limitation. Preclinical studies suggest that benzodiazepines may interfere with synaptogenesis and neuronal survival during critical periods of brain development, raising concern about possible long-term neurocognitive consequences [26]. A recent Cochrane review found the available evidence insufficient to clearly establish the benefits or harms of midazolam compared with placebo, opioids, or non-pharmacological strategies, and emphasized the lack of robust long-term neurodevelopmental safety data [1].

Observational studies have also raised concerns regarding prolonged benzodiazepine exposure in extremely preterm infants. In infants exposed to benzodiazepines, often in combination with opioids, for more than seven days, lower neurodevelopmental scores at two years of age have been reported [26]. Similarly, meta-analytic data suggest that although intravenous midazolam may increase sedation, it may also be associated with adverse neurological outcomes, including intraventricular hemorrhage or periventricular leukomalacia, and longer NICU stay [20]. Therefore, midazolam should not be considered a first-line routine analgosedation agent for all neonates. Its use should be restricted to selected clinical situations, administered for the shortest possible duration, and guided by continuous clinical reassessment.

### 4.3. Alpha-2 Adrenergic Agonists

Dexmedetomidine is an alpha-2 adrenergic receptor agonist with sedative, anxiolytic, and analgesic-sparing properties [14]. Unlike benzodiazepines, it produces a sleep-like sedation that more closely resembles physiological sleep and is associated with minimal respiratory depression. This characteristic is particularly attractive in neonates because spontaneous breathing and upper airway tone are generally preserved [14]. Dexmedetomidine may therefore be useful in selected infants requiring analgosedation without significant suppression of respiratory drive.

Interest in dexmedetomidine has increased because it may reduce exposure to opioids and benzodiazepines. A systematic review by Portelli and colleagues, including six studies and 252 neonates, suggested that dexmedetomidine may be effective for analgosedation in this population, with no major safety signals identified in the available studies [16]. Observational data also suggest that dexmedetomidine, either alone or as an adjunct to opioids or benzodiazepines, may decrease cumulative exposure to these agents and facilitate ventilator weaning or extubation in selected neonates [27].

The main reported adverse effects of dexmedetomidine are bradycardia and hypotension [23]. These events are often mild or clinically manageable, but they require close monitoring, especially in preterm infants, neonates with hemodynamic instability, and those receiving other cardiovascularly active drugs [27]. Because dexmedetomidine undergoes hepatic metabolism, dose adjustment and careful titration may be required in neonates with impaired hepatic function or during conditions that alter drug clearance.

Despite its increasing use, evidence supporting dexmedetomidine for neonatal analgosedation remains limited. Data on pharmacokinetics, pharmacodynamics, optimal dosing, safety in extremely preterm infants, and long-term neurodevelopmental outcomes are still insufficient [27]. Further randomized controlled trials are needed to clarify whether dexmedetomidine can safely replace or reduce the use of opioids and benzodiazepines in different neonatal populations. Ongoing studies comparing dexmedetomidine with fentanyl, morphine, or ketamine-based regimens may help define its future role in NICU analgosedation [28]. Therefore, current evidence supports cautious and protocolized use of dexmedetomidine in selected neonates, but its role in extremely preterm infants remains incompletely defined because pharmacokinetic, dosing, and long-term neurodevelopmental data are still limited.

Although dexmedetomidine is increasingly used in NICUs, several clinically important knowledge gaps remain. First, pharmacokinetic data in extremely preterm neonates are still insufficient. The available phase II/III multicenter pharmacokinetic study included preterm and term neonates from 28 to 44 weeks of gestational age and showed that preterm neonates had reduced plasma clearance and a longer elimination half-life than term neonates, supporting the need for cautious dose titration in immature infants [29]. However, robust pharmacokinetic and pharmacodynamic data for extremely preterm infants below 28 weeks’ gestation remain limited, particularly during prolonged infusions, critical illness, therapeutic hypothermia, hepatic dysfunction, or concomitant exposure to opioids, benzodiazepines, or vasoactive drugs. Because dexmedetomidine undergoes extensive hepatic metabolism, developmental immaturity of glucuronidation and cytochrome P450 pathways may contribute to delayed clearance, drug accumulation, bradycardia, hypotension, and prolonged sedative effects in the most immature neonates [30].

Second, long-term neurodevelopmental safety has not been definitively established. Observational data in very preterm infants are encouraging but remain limited by retrospective design, small sample size, confounding by illness severity, and differences in concomitant sedative exposure. Similarly, in encephalopathic neonates undergoing therapeutic hypothermia, a recent retrospective cohort comparing dexmedetomidine with morphine found comparable Bayley Scales of Infant Development outcomes at 18–24 months, with some early 12-month motor scores favoring dexmedetomidine; however, these findings require confirmation in larger prospective studies [31]. Therefore, dexmedetomidine should currently be considered a promising opioid- and benzodiazepine-sparing agent rather than a medication with fully established long-term neurodevelopmental safety in neonates.

Third, dosing practices vary substantially among NICUs. Dexmedetomidine remains off-label in neonates, and published studies and institutional protocols differ with respect to loading-dose use, starting dose, titration interval, maximum infusion rate, duration of therapy, weaning strategy, and transition to clonidine. Reported infusion ranges commonly begin at approximately 0.1–0.3 mcg/kg/h and are titrated according to sedation scores and hemodynamic tolerance, but higher maximum doses, loading-dose practices, and tapering approaches differ between centers [32]. Until more robust neonatal pharmacokinetic, pharmacodynamic, and long-term safety data are available, dexmedetomidine should be used within unit-specific protocols that include gestational-age–appropriate dosing, avoidance of unnecessary loading doses in hemodynamically fragile infants, continuous heart-rate and blood-pressure monitoring, serial pain and analgosedation assessment, and structured weaning to reduce rebound agitation or withdrawal.

### 4.4. Ketamine

Ketamine is a phencyclidine derivative that produces dissociative anesthesia through non-competitive antagonism of the NMDA receptor [33]. NMDA receptors are involved in excitatory neurotransmission, synaptic plasticity, central sensitization, and neuronal survival. By inhibiting NMDA-mediated calcium influx, ketamine provides analgosedation, amnesia, and dissociation. It also interacts with opioid receptors, monoaminergic pathways, and voltage-gated calcium channels, contributing to its multimodal analgesic and sedative profile [33].

A distinctive feature of ketamine is its relative preservation of airway reflexes and cardiovascular stability [33]. Through sympathetic stimulation, it may increase heart rate, systemic blood pressure, and cardiac output. These properties make ketamine potentially useful in selected hemodynamically unstable neonates or during painful procedures when preservation of cardiovascular tone is desirable. However, this same sympathetic effect may be undesirable in infants with certain cardiac conditions, pulmonary hypertension, or limited myocardial reserve.

Neonatal ketamine pharmacokinetics are variable and incompletely defined. The drug is metabolized mainly in the liver through CYP2B6 and CYP3A4 pathways to norketamine, an active metabolite with analgesic properties, and is subsequently eliminated by the kidneys [33]. Immature hepatic metabolism, altered protein binding, and developmental differences in body composition may prolong elimination and increase interindividual variability in neonates.

Concerns about ketamine exposure during early development derive largely from experimental studies. Repeated or high-dose ketamine exposure has been associated with neuroapoptosis in the developing brain, possibly related to NMDA receptor blockade during periods of intense synaptogenesis [33]. Conversely, ketamine may exert neuroprotective effects in specific contexts, such as hypoxia or inflammatory stress, by reducing excitotoxic glutamate release and modulating inflammatory pathways [34]. This dual profile highlights the complexity of translating preclinical findings into neonatal clinical practice.

Overall, ketamine may offer important advantages for selected procedures or clinical conditions, especially when rapid analgosedation is required in infants at risk of hemodynamic instability. Nevertheless, its use should remain cautious and individualized, particularly when repeated or prolonged administration is considered. Further neonatal studies are needed to define optimal dosing, safety margins, and long-term neurodevelopmental outcomes.

### 4.5. Additional Agents Used in Selected Neonatal Intensive Care Unit (NICU) Settings

Although morphine, fentanyl, midazolam, dexmedetomidine, and ketamine are among the most frequently discussed agents for neonatal analgosedation, other drugs may be used in selected NICU contexts, including remifentanil, clonidine, and paracetamol/acetaminophen. Their use is generally more specific and should be guided by local protocols, gestational age, organ maturity, hemodynamic status, expected duration of therapy, and the type and intensity of pain.

Remifentanil is an ultra-short-acting synthetic opioid metabolized by nonspecific plasma and tissue esterases, resulting in rapid onset and offset that are relatively independent of hepatic and renal maturation. These properties make it attractive for brief but highly painful procedures, particularly endotracheal intubation, short procedural sedation, and selected perioperative situations [35]. A systematic review of fetal, preterm, and term neonatal exposure to remifentanil reported potential usefulness for neonatal procedural pain and invasive procedures, but also emphasized the need for careful monitoring because respiratory depression, chest wall rigidity, bradycardia, and hemodynamic instability may occur [35]. Retrospective neonatal data suggest that remifentanil can provide effective premedication for elective intubation, but dosing varies and administration should occur only with continuous cardiorespiratory monitoring and personnel prepared for airway and ventilation support [36]. Because of its very short context-sensitive half-life, remifentanil is not generally suitable as a sole agent for sustained postoperative pain or prolonged NICU analgosedation unless followed by an alternative analgesic strategy.

Clonidine is an alpha-2 adrenergic agonist with analgosedation-sparing, sympatholytic, and anti-withdrawal properties. Compared with dexmedetomidine, clonidine has a longer half-life and is available for enteral administration, which may make it useful as an adjunct during weaning from prolonged opioid or sedative therapy, as part of withdrawal management, or as an analgosedation-sparing agent in selected neonates [37]. Neonatal formularies and institutional guidance describe its use for analgosedation in mechanically ventilated infants and for neonatal abstinence or iatrogenic withdrawal, with recommendations for heart-rate, blood-pressure, oxygen-saturation, and pain/comfort-score monitoring [37]. However, robust neonatal randomized data remain limited, and adverse effects such as hypotension, bradycardia, oversedation, and rebound hypertension or rebound withdrawal after abrupt discontinuation should be considered [37]. Therefore, clonidine should be introduced cautiously, titrated gradually, and tapered rather than stopped abruptly, especially after prolonged administration.

Paracetamol/acetaminophen is a non-opioid analgesic increasingly used for mild to moderate pain and as part of multimodal postoperative analgesia. It has minimal respiratory depressant effects and may reduce opioid requirements in selected postoperative neonates, although it is not an adequate sole agent for severe pain [38,39,40]. A Cochrane review found limited evidence supporting paracetamol for neonatal pain prevention or treatment and emphasized uncertainty regarding efficacy for several procedural pain indications [38]. Subsequent reviews suggest that paracetamol may have opioid-sparing effects for some major pain syndromes and postoperative settings, but it is generally ineffective as a standalone intervention for acute procedural pain such as heel lance or venipuncture [39]. Intravenous acetaminophen pharmacokinetic studies have provided dosing and safety data in neonates, but clinicians should still consider gestational age, postnatal age, hepatic function, total daily dose, and concomitant hepatotoxic exposures [40]. In practice, paracetamol/acetaminophen is best considered an adjunct within multimodal analgosedation, particularly when the goal is to reduce cumulative opioid exposure while maintaining adequate pain control.

Overall, remifentanil, clonidine, and paracetamol/acetaminophen may broaden the therapeutic options for neonatal analgosedation, but none should be considered universally applicable. Remifentanil may be useful for short painful procedures requiring rapid recovery; clonidine may have a role as an adjunctive analgosedation-sparing or withdrawal-management agent; and paracetamol/acetaminophen may contribute to opioid-sparing multimodal analgosedation, especially for mild to moderate or postoperative pain. Their use should remain individualized and protocol-based, with careful monitoring for drug-specific adverse effects.

### 4.6. General Considerations

The pharmacological management of pain and analgosedation in the NICU requires a balanced approach. Undertreated pain may contribute to physiological instability, altered stress responses, pain sensitization, and adverse neurodevelopmental outcomes. Conversely, unnecessary or prolonged exposure to analgosedation agents may increase the risk of respiratory depression, hypotension, feeding intolerance, tolerance, withdrawal, and possible neurodevelopmental effects. For this reason, analgosedation should be based on validated assessment tools, clear clinical indications, regular reassessment, and the lowest effective dose for the shortest appropriate duration.

Whenever possible, pharmacological strategies should be integrated with non-pharmacological interventions, such as facilitated tucking, non-nutritive sucking, breastfeeding, oral sucrose, skin-to-skin care, swaddling, and environmental modulation. A multimodal approach may reduce drug requirements while improving comfort and physiological stability.

Neurodevelopmental concerns related to neonatal analgosedation should be interpreted in light of both randomized and observational evidence. Cohort studies in extremely preterm and very preterm infants have reported associations between greater neonatal exposure to opioids and/or benzodiazepines and adverse neurodevelopmental outcomes, including lower cognitive, motor, or language scores at 18–24 months and during childhood follow-up [41,42,43,44,45,46]. For example, a large cohort study of extremely preterm infants found that opioid and/or benzodiazepine exposure during NICU hospitalization was associated with worse 2-year neurodevelopmental outcomes, although the authors emphasized the difficulty of separating medication effects from illness severity, painful procedures, mechanical ventilation, and other neonatal morbidities [41]. Other observational cohorts have reported associations between morphine exposure and altered brain growth or poorer neurodevelopmental outcomes, including smaller cerebellar growth and lower developmental scores at 18 months [42], while longer-term follow-up studies have raised concerns regarding cognitive, behavioral, and socioemotional outcomes at 5 years or school age [43,44]. Recent data also suggest that neonatal opioid exposure may be associated with differences in early childhood neurodevelopment and brain development among children born very preterm, particularly with longer duration or higher cumulative exposure [45,46].

However, causality remains uncertain. Infants receiving opioids, benzodiazepines, or prolonged analgosedation are often the most immature and critically ill, have greater exposure to invasive ventilation and painful procedures, and are at higher baseline risk for brain injury and adverse neurodevelopment [41,45,46]. These confounders may contribute substantially to the observed associations. In addition, untreated or repeated pain itself has been linked to altered brain development, stress regulation, and later neurodevelopmental outcomes, making avoidance of analgosedation an inappropriate strategy [47,48]. Current evidence therefore supports neither routine prolonged analgosedation nor withholding analgosedation when pain is present. Instead, the available data support individualized treatment guided by validated pain and analgosedation scores, use of the lowest effective dose for the shortest clinically appropriate duration, integration of non-pharmacological strategies, avoidance of unnecessary combined opioid–benzodiazepine exposure, and long-term neurodevelopmental follow-up in infants requiring prolonged or high cumulative analgosedation therapy.

## 5. Non-Pharmacological Strategies

Non-pharmacological interventions are an essential component of neonatal pain management and should be integrated into routine NICU care whenever feasible [1]. These strategies are particularly useful for preventing or reducing pain associated with brief procedures, limiting stress responses, improving physiological stability, and decreasing the need for pharmacological agents. Their use is especially relevant in preterm and medically fragile neonates, in whom drug exposure may be associated with respiratory, hemodynamic, gastrointestinal, and neurodevelopmental concerns. Non-pharmacological approaches are not intended to replace pharmacological analgosedation when moderate or severe pain is expected, but they may provide meaningful additive benefits as part of a multimodal pain-management plan.

Non-pharmacological interventions can be classified in several ways, and terminology varies across studies and guidelines. In this review, we use the terms “proximal” and “distal” as a conceptual framework previously described in the neonatal pain literature, rather than as a new classification created by the authors. Bucsea and Pillai Riddell categorized evidence-based non-pharmacological pain-management approaches for hospitalized neonates into proximal, distal, and procedural methods [49]. In this framework, proximal strategies refer to interventions involving direct physical, tactile, oral, postural, or parental contact with the infant, such as skin-to-skin care, breastfeeding, non-nutritive sucking, facilitated tucking, swaddling, and oral sucrose. Distal strategies refer to interventions that modify the infant’s sensory environment, including regulation of light, noise, music, maternal voice, odor, and other environmental stimuli. Because these terms are less commonly used in clinical guidelines than broader terms such as physical, behavioral, feeding-related, and environmental interventions, they should be interpreted as an organizational framework intended to distinguish direct contact-based interventions from environmental modulation strategies. Accordingly, in the following sections, proximal strategies are discussed first, followed by distal environmental strategies. The principal non-pharmacological interventions used in the NICU are summarized in Table 2.

### 5.1. Proximal Pain Management Methods

Proximal pain-management strategies support neonatal self-regulation and help infants return to baseline after painful or stressful procedures. They usually involve comforting tactile, oral, or postural input delivered before, during, or immediately after a noxious stimulus. From the perspective of Gate Control Theory, non-noxious sensory stimulation may modulate nociceptive transmission and reduce pain perception by activating inhibitory mechanisms within the somatosensory pathways [50,51]. In neonates, these interventions may also promote behavioral organization, autonomic stability, and parent–infant bonding.

Oral sucrose is one of the most extensively studied non-pharmacological interventions for procedural pain in neonates. It has demonstrated efficacy in reducing behavioral pain responses during brief invasive procedures, including heel lance, venipuncture, and intramuscular injections, in both preterm and term infants [52]. Its analgesic effect is thought to involve activation of endogenous opioid pathways, particularly when combined with non-nutritive sucking. No major serious adverse effects have been consistently reported; however, uncertainty remains regarding the optimal dose, frequency of administration, and safety of repeated exposure, especially in extremely preterm, clinically unstable, or mechanically ventilated infants. Moderate-quality evidence suggests that combining sucrose with interventions such as non-nutritive sucking may be more effective than sucrose alone [52].

Skin-to-skin care, also known as kangaroo care, is another well-established proximal intervention [53]. By placing the infant directly on the parent’s chest, this approach provides warmth, containment, familiar sensory input, and emotional regulation. Skin-to-skin care has been shown to reduce behavioral and physiological pain responses compared with standard care, particularly during minor procedures [53]. Despite its effectiveness, implementation in NICUs may be limited by workload, clinical instability, space constraints, and competing care priorities. Facilitating parental presence during acute procedures may improve the feasibility and consistency of this intervention [54].

Other containment-based strategies include facilitated tucking, swaddling, and supported positioning. These techniques involve maintaining the infant in a flexed, midline, developmentally supportive posture, thereby reducing motor disorganization and promoting self-soothing [55]. They are simple, low-cost, and easily applicable during routine procedures, either alone or in combination with sucrose, non-nutritive sucking, or parental contact.

Breastfeeding provides multimodal analgesia through the combined effects of sweet taste, sucking, maternal contact, warmth, odor, and emotional comfort. In term neonates, breastfeeding has demonstrated greater analgesic efficacy than some single interventions such as kangaroo care or swaddling [56]. The administration of expressed breast milk by syringe appears less effective, likely because it lacks the combined tactile, olfactory, sucking, and relational components of direct breastfeeding [57]. When direct breastfeeding is not feasible, expressed breast milk may still be considered as part of a broader comfort strategy, although its analgesic effect may be weaker than that of sucrose.

Co-bedding, defined as placing twins or multiples in the same incubator, has also been explored as a proximity-based intervention. Available evidence suggests that co-bedding may support stress regulation after acute procedures, as reflected by lower cortisol concentrations and faster recovery of physiological stability [55]. However, its use should be guided by local safety protocols, infection-control policies, and careful clinical assessment.

### 5.2. Distal Pain Management Methods

Distal pain-management strategies focus on modifying the NICU environment to reduce overstimulation, distress, and pain reactivity. The NICU exposes neonates to repeated sensory stressors, including bright light, excessive noise, alarms, handling, unfamiliar odors, and disrupted sleep–wake rhythms. Environmental regulation may help improve autonomic stability, sleep organization, feeding tolerance, and recovery after painful procedures [57].

Light modulation has been investigated as a potential supportive intervention. High-intensity lighting may contribute to physiological stress, whereas cycled or reduced lighting has been associated with improved heart-rate stability, circadian organization, sleep patterns, and feeding outcomes. Some studies suggest that covering infants’ eyes may attenuate behavioral pain responses when applied after painful procedures, although effects on physiological parameters such as heart rate and oxygen saturation are less consistent [58]. These findings indicate that light reduction may support comfort but should not be considered a standalone analgesic intervention.

Auditory interventions, including music therapy, lullabies, recorded maternal voice, and intrauterine-like sounds, have also been studied in neonatal pain management. These approaches may reduce arousal and behavioral signs of distress, although results vary according to intervention type, timing, gestational age, and clinical condition [59]. Among auditory interventions, exposure to the maternal voice appears particularly promising and has shown analgesic effects in both preterm and term infants [60,61]. Classical music has been associated with reduced pain responses during routine procedures such as heel lancing [62], while lullabies or music familiar from the prenatal period may improve behavioral indicators of pain even when physiological responses remain unchanged [63]. Rhythmic sounds resembling the intrauterine environment, such as maternal heartbeat recordings, may also contribute to reduced pain reactivity and improved comfort [64].

Olfactory stimulation represents another emerging distal strategy [1]. Familiar scents, particularly maternal odor, may have calming effects and may support neonatal regulation during and after stressful procedures. However, evidence remains limited, and further studies are needed to determine optimal timing, safety, and clinical effectiveness.

Overall, non-pharmacological interventions are most effective when applied consistently, early, and in combination. Multimodal strategies, such as sucrose plus non-nutritive sucking, skin-to-skin care plus breastfeeding, or facilitated tucking combined with environmental control, may provide greater analgesic benefit than isolated interventions. Their implementation requires staff education, parental involvement, and protocol-driven integration into routine NICU procedures [65,66].

## 6. Monitoring and Assessment

Pain and sedation assessment is a prerequisite for safe and effective analgosedation in the NICU. Unlike older children and adults, neonates cannot verbally express pain or discomfort; therefore, evaluation relies on structured observation of behavioral, physiological, and contextual indicators [6]. Facial expression, crying or irritability, body movements, muscle tone, behavioral state, heart rate, oxygen saturation, respiratory pattern, blood pressure, and autonomic instability may all contribute to pain assessment. However, none of these parameters is specific to pain when considered in isolation. For this reason, validated multidimensional tools are essential to improve consistency, guide treatment decisions, and reduce both undertreatment of pain and excessive sedation.

An ideal neonatal pain and analgosedation assessment tool should be reliable, reproducible, easy to apply at the bedside, sensitive to changes over time, and adaptable to different gestational ages, clinical conditions, and types of pain exposure. In preterm or critically ill neonates, assessment is particularly challenging because behavioral responses may be blunted by immaturity, neurological injury, severe illness, mechanical ventilation, or the use of sedative or neuromuscular-blocking agents. Pain scores should therefore always be interpreted within the broader clinical context and combined with repeated bedside evaluation.

Several validated instruments have been developed to assess pain, agitation, and analgosedation in neonates [67,68,69]. Among the most frequently used are the COMFORTneo scale, the Neonatal Pain, Agitation and Sedation Scale (N-PASS), and the Premature Infant Pain Profile-Revised (PIPP-R). These scales differ in their intended use: some are better suited for prolonged pain and analgosedation monitoring, whereas others are designed primarily for acute procedural pain.

The COMFORTneo scale is one of the most widely validated instruments for assessing prolonged pain, distress, and analgosedation in neonates admitted to intensive care. It was adapted from the original COMFORT scale used in pediatric intensive care and modified to improve applicability in both preterm and term neonates [69]. The tool evaluates behavioral domains such as alertness, calmness or agitation, respiratory response or crying, body movement, facial tension, and muscle tone. These items provide a multidimensional estimate of neonatal comfort and distress. A major strength of COMFORTneo is its usefulness for repeated assessment in infants receiving prolonged opioid or sedative therapy, especially mechanically ventilated neonates. It can help clinicians titrate analgosedation drugs, identify insufficient pain control, and avoid excessive analgosedation. Because subtle behavioral changes may precede overt physiological deterioration, systematic scoring can support earlier therapeutic adjustment. However, interpretation may be limited in infants with severe neurological impairment, extreme prematurity, deep sedation, or neuromuscular blockade. Adequate staff training and standardized implementation protocols are essential to ensure interobserver reliability and consistent clinical use.

The N-PASS was developed to assess pain, agitation, and analgosedation across a broad range of neonatal populations, including ventilated and non-ventilated infants [68]. It includes five domains: crying or irritability, behavioral state, facial expression, extremity tone, and vital signs. Positive scores indicate increasing pain or agitation, whereas negative scores may indicate deeper analgosedation. One of the main advantages of N-PASS is that it evaluates both discomfort and sedation depth within the same instrument. This is particularly valuable in critically ill neonates receiving continuous analgosedation infusions, where clinicians must balance adequate comfort against the risks of oversedation. The scale also includes gestational age adjustment, which is important because extremely preterm infants may show attenuated behavioral responses despite clinically relevant nociceptive exposure. In routine practice, serial N-PASS assessment may guide individualized titration of opioids, benzodiazepines, and other analgosedation agents, potentially reducing unnecessary drug exposure while maintaining adequate analgesia. Nevertheless, N-PASS has limitations. Physiological variables may be affected by conditions unrelated to pain, including sepsis, hypoxemia, respiratory distress, hemodynamic instability, or neurological disease. In neonates with encephalopathy, severe prematurity, or altered tone, distinguishing agitation, pain, and neurological dysfunction may be difficult. For this reason, N-PASS results should be integrated with clinical judgment and ongoing reassessment.

The PIPP-R is a validated instrument primarily designed for assessment of acute procedural pain in preterm and term neonates [69]. It incorporates behavioral, physiological, and contextual indicators, including facial actions such as brow bulge, eye squeeze, and nasolabial furrow, together with changes in heart rate and oxygen saturation. Gestational age and behavioral state are included in the scoring system to account for developmental differences in pain expression. PIPP-R is particularly useful for short painful procedures such as heel lance, venipuncture, arterial puncture, endotracheal suctioning, and catheter placement. Its incorporation of gestational age is a major advantage, since very preterm neonates may display limited behavioral responses even when exposed to painful stimuli. The scale has demonstrated good validity and reliability and is frequently used in both clinical research and bedside practice to evaluate the effectiveness of pharmacological and non-pharmacological interventions. However, PIPP-R is not designed for continuous monitoring of prolonged pain or analgosedation. It should therefore not be used as the sole assessment tool in infants receiving long-term opioid or analgosedation infusions, during mechanical ventilation, or in situations where ongoing distress and sedation depth must be monitored over time. In these contexts, tools such as COMFORTneo or N-PASS may be more appropriate.

Overall, the choice of assessment instrument should depend on the clinical setting, the type of pain, and the intended therapeutic decision. Procedural pain, postoperative pain, prolonged ventilation, and continuous analgosedation infusion may require different tools or complementary approaches. Regardless of the scale selected, routine assessment should be embedded in a protocol-driven strategy that links pain and analgosedation scores to specific interventions, reassessment intervals, and weaning plans.

## 7. Monitoring for Adverse Effects During Analgosedation

Although analgosedation is a fundamental component of neonatal intensive care, pharmacological treatment may cause clinically significant adverse effects. Neonates are particularly vulnerable because of immature drug metabolism, reduced clearance, altered protein binding, developmental differences in receptor sensitivity, and limited physiological reserve [1]. Continuous monitoring is therefore mandatory, especially in preterm infants and in neonates receiving prolonged opioid, benzodiazepine, or combined sedative infusions.

Monitoring should include repeated assessment of respiratory status, hemodynamics, gastrointestinal tolerance, neurological state, depth of sedation, cumulative drug exposure, and signs of tolerance or withdrawal [1]. The objective is to maintain adequate comfort while using the lowest effective dose for the shortest appropriate duration.

Respiratory depression is one of the most important adverse effects associated with opioid and sedative administration in neonates [7,70]. Morphine and fentanyl may reduce respiratory drive, blunt ventilatory responses to hypercapnia, and increase the risk of apnea, particularly in preterm infants. The risk is greater when opioids are combined with benzodiazepines or other analgosedation agents. Continuous cardiorespiratory monitoring, pulse oximetry, and careful assessment of respiratory rate, apnea frequency, blood gases, ventilator parameters, and extubation readiness are therefore essential. Clinicians must balance adequate analgesia with preservation of spontaneous respiratory function and avoidance of unnecessary prolongation of mechanical ventilation.

Analgosedation agents may compromise cardiovascular stability through vasodilation, myocardial depression, altered autonomic tone, or reduced sympathetic response. Morphine has been associated with hypotension in preterm infants, particularly after rapid administration or in neonates with limited hemodynamic reserve [7,70]. Hypotension is clinically relevant because it may reduce systemic and cerebral perfusion, especially in extremely preterm infants with impaired autoregulation. Blood pressure, heart rate, capillary refill, urine output, lactate levels, and the need for vasoactive support should be monitored closely. Dose titration should be individualized, particularly in infants with sepsis, patent ductus arteriosus, pulmonary hypertension, hypoxic–ischemic encephalopathy, or severe respiratory failure.

Opioids can reduce gastrointestinal motility by inhibiting enteric nervous system activity [47]. Prolonged exposure may lead to delayed gastric emptying, abdominal distension, feeding intolerance, constipation, and paralytic ileus. These effects may delay advancement of enteral nutrition, prolong parenteral nutrition, and increase the risk of cholestasis, catheter-related infection, and impaired growth. During extended opioid treatment, clinicians should monitor abdominal examination findings, stool frequency, gastric residuals where routinely assessed, emesis, abdominal radiographic findings when indicated, and progression of enteral feeding. Reducing opioid exposure through multimodal analgosedation and regular reassessment may help limit gastrointestinal complications.

Tolerance is a frequent challenge in neonates exposed to prolonged analgosedation infusions [71]. Repeated receptor stimulation may progressively reduce drug effect, leading to escalating dose requirements to achieve the same level of analgosedation. This is particularly relevant in mechanically ventilated infants receiving continuous opioid infusions over several days. Before increasing doses, clinicians should reassess whether agitation is due to pain, ventilator asynchrony, hunger, environmental stress, hypoxemia, withdrawal, neurological disease, or progression of the underlying illness. Strategies to reduce tolerance include avoiding unnecessary continuous infusions, using intermittent dosing when appropriate, integrating non-pharmacological interventions, applying validated pain and sedation scales, and implementing daily reassessment of ongoing drug need.

Validated withdrawal assessment instruments are useful to standardize recognition of iatrogenic withdrawal, reduce interobserver variability, and link clinical findings to weaning or rescue-treatment decisions. The Finnegan Neonatal Abstinence Scoring System was originally developed to quantify neonatal withdrawal signs, particularly in opioid-exposed newborns, by systematically scoring central nervous system, gastrointestinal, autonomic, and respiratory manifestations [72]. Modified Finnegan-based scores remain widely used in neonatal units and may be helpful when withdrawal symptoms emerge during or after opioid weaning; however, they were primarily designed for neonatal abstinence syndrome related to prenatal drug exposure and may be less specific for iatrogenic withdrawal in critically ill neonates receiving analgosedation infusions [73,74]. For infants and children exposed to prolonged opioids and/or benzodiazepines in intensive care, the Withdrawal Assessment Tool-1 (WAT-1) provides a structured approach to monitoring iatrogenic withdrawal, including gastrointestinal symptoms, autonomic signs, motor findings, behavioral state, and response to stimulation [47]. WAT-1 has shown validity and generalizability in critically ill pediatric populations, but neonatal-specific validation remains more limited; therefore, its application in NICU patients should be interpreted in relation to gestational age, illness severity, neurological status, and local protocol experience [75]. In practice, withdrawal monitoring should begin when prolonged opioid or benzodiazepine therapy is being tapered, particularly after continuous or scheduled exposure for several days, and should continue until symptoms resolve and treatment has been discontinued. Whichever tool is selected, consistent staff training, predefined scoring intervals, and protocolized responses to rising scores are essential to improve reliability and prevent both undertreatment of withdrawal and unnecessary prolongation of sedative or analgesic exposure.

Gradual tapering protocols are recommended for neonates receiving prolonged analgosedation [1,76]. The rate of dose reduction should be individualized according to the duration of treatment, cumulative exposure, current clinical stability, and withdrawal symptoms. Structured withdrawal assessment tools, including modified Finnegan-based scores or unit-specific neonatal withdrawal scales, may help standardize monitoring and guide weaning. Early recognition is important because untreated withdrawal can increase physiological stress, impair feeding and growth, delay recovery, and prolong hospitalization.

In summary, adverse-effect monitoring should be considered an integral part of neonatal analgosedation rather than a separate safety measure. A protocol-driven, multidisciplinary approach involving neonatologists, nurses, pharmacists, respiratory therapists, and, when appropriate, pain specialists can improve consistency of assessment, reduce unnecessary drug exposure, and support safer individualized care.

## 8. Future Perspectives in Neonatal Pain Assessment and Analgosedation

Despite substantial progress in the recognition and management of neonatal pain, accurate pain assessment in the NICU remains challenging. Current tools are largely based on behavioral and physiological indicators that require observer interpretation and may be influenced by gestational age, illness severity, neurological status, mechanical ventilation, and exposure to analgosedation drugs. Extremely preterm or critically ill neonates may show blunted behavioral responses despite significant nociceptive stimulation, whereas physiological changes such as tachycardia, desaturation, or blood pressure instability may reflect conditions other than pain. These limitations highlight the need for more objective, continuous, and developmentally sensitive methods of pain assessment.

Emerging technologies may help improve the precision and reproducibility of neonatal pain evaluation. Automated facial-expression analysis, heart rate variability assessment, skin conductance monitoring, electroencephalographic biomarkers, and near-infrared spectroscopy have all been investigated as potential tools to quantify pain-related responses in newborns [76,77]. These methods may provide complementary information on autonomic, cortical, and behavioral responses to noxious stimuli. In particular, cerebral monitoring techniques could help identify pain-related cortical activation in infants whose external behavioral responses are limited by prematurity, illness, or pharmacological analgosedation.

Artificial intelligence and machine-learning systems may further support neonatal pain assessment by integrating multiple data streams, including facial activity, vital signs, oxygenation patterns, movement, sleep–wake state, and cerebral monitoring [50]. In the future, such systems could enable continuous real-time assessment of pain, agitation, and sedation depth, allowing earlier recognition of distress and more precise titration of analgosedation therapies. However, before implementation in routine NICU care, these technologies require rigorous validation across diverse neonatal populations, including extremely preterm infants, term neonates with encephalopathy, postoperative patients, and infants receiving mechanical ventilation or therapeutic hypothermia.

Future research should also focus on optimizing individualized analgosedation strategies. Developmental pharmacology, pharmacogenomics, and precision-medicine approaches may help clarify why neonates differ substantially in drug response, efficacy, tolerance, withdrawal risk, and adverse effects. Better understanding of maturational changes in hepatic metabolism, renal clearance, receptor expression, and blood–brain barrier permeability could support more accurate dosing strategies and reduce unnecessary drug exposure. Long-term follow-up studies are particularly needed to determine how neonatal exposure to opioids, benzodiazepines, alpha-2 agonists, ketamine, and multimodal analgosedation protocols may influence neurodevelopmental outcomes.

At the same time, technological innovation should not replace structured clinical assessment. Validated bedside instruments such as COMFORTneo, N-PASS, and PIPP-R remain essential for daily practice, each offering specific advantages according to the clinical context, from acute procedural pain to prolonged analgosedation monitoring. Future models of care will likely combine standardized observational scales with objective physiological and neurophysiological measures, creating a more comprehensive and individualized approach to neonatal pain management.

A multidisciplinary, protocol-driven strategy remains fundamental. Neonatologists, nurses, pharmacists, respiratory therapists, developmental-care specialists, and parents all play important roles in recognizing pain, applying non-pharmacological interventions, titrating pharmacological therapies, and monitoring adverse effects. Respiratory depression, hypotension, gastrointestinal dysmotility, tolerance, and iatrogenic withdrawal remain clinically relevant complications that require continuous surveillance and individualized management.

In conclusion, the future of neonatal pain assessment and analgosedation lies in the integration of validated clinical tools, emerging monitoring technologies, precision pharmacology, and family-centered developmental care. Continued research should aim to improve pain recognition, minimize both untreated pain and unnecessary drug exposure, and ultimately promote better short- and long-term outcomes for critically ill newborns.

## 9. Conclusions

Pain prevention and control are essential components of high-quality neonatal intensive care. Preterm and critically ill newborns are repeatedly exposed to invasive procedures, mechanical ventilation, postoperative discomfort, and advanced life-support interventions, all of which may generate acute and prolonged pain. Because untreated pain can contribute to physiological instability, altered stress regulation, pain sensitization, and adverse neurodevelopmental outcomes, effective analgosedation should be considered a core element of neonatal care rather than an optional supportive measure.

At the same time, analgosedation therapy in neonates requires particular caution. Developmental immaturity, organ dysfunction, altered receptor sensitivity, and wide pharmacokinetic variability make this population highly vulnerable to both under-treatment and over-treatment. Inadequate analgesia may worsen cardiorespiratory instability and stress responses, whereas excessive or prolonged drug exposure may increase the risk of respiratory depression, hypotension, gastrointestinal dysmotility, tolerance, withdrawal syndrome, and potential effects on the developing brain.

A rational approach to neonatal analgosedation should therefore be individualized, multimodal, and continuously reassessed. Pharmacological treatment should be guided by clear clinical indications, gestational and postnatal age, clinical severity, organ function, and expected duration of painful exposure. Opioids, benzodiazepines, dexmedetomidine, ketamine, and other agents may all have a role in selected situations, but their use should be based on careful titration, validated pain and analgosedation scores, and close monitoring for adverse effects.

Non-pharmacological interventions are equally important and should be systematically incorporated into NICU practice. Oral sucrose, non-nutritive sucking, facilitated tucking, swaddling, breastfeeding, skin-to-skin care, parental involvement, and environmental modulation can reduce procedural pain and stress, particularly when used in combination. These strategies may enhance comfort, support physiological stability, and reduce unnecessary pharmacological exposure.

Validated assessment tools, including COMFORTneo, N-PASS, and PIPP-R, remain central to safe pain and analgosedation management. Their routine use promotes standardized evaluation, supports therapeutic decision-making, and helps clinicians identify inadequate analgesia, excessive sedation, or evolving withdrawal. Future advances, including automated pain-recognition systems, neurophysiological monitoring, artificial intelligence, pharmacogenomics, and precision-medicine approaches, may further improve the objectivity and personalization of neonatal pain management.

The goal of analgosedation in the NICU is to achieve effective pain relief, adequate comfort, and clinical stability while minimizing avoidable drug exposure and treatment-related complications. A protocol-driven, evidence-informed, multidisciplinary, and family-centered approach offers the best opportunity to improve immediate care and support healthier long-term outcomes for vulnerable newborns.

## Figures and Tables

**Table 1:** Main pharmacological agents used for analgosedation in the Neonatal Intensive Care Unit (NICU).

Drug Class	Examples	Notes	Dosage
Opioids	Morphine, Fentanyl	May cause hypotension, tolerance, withdrawal; morphine has slower onset [10]	Morphine: 10–30 mcg/kg/h infusion; Fentanyl: 1–2 mcg/kg bolus or 0.5–2 mcg/kg/h infusion
Benzodiazepines	Midazolam, Lorazepam	Sedative only; risk of hypotension, myoclonus, neurotoxicity [11]	Midazolam: 30–60 mcg/kg/h infusion; Lorazepam: 0.05–0.1 mg/kg/dose
Alpha-2 Agonists	Dexmedetomidine	Minimal respiratory depression; neuroprotective potential [12]	Dexmedetomidine: 0.1–1 mcg/kg/h infusion
N-methyl-D-aspartate (NMDA) Antagonists	Ketamine	Rapid onset; cardiovascular stability; potential neurotoxicity in high doses [13]	Ketamine: 0.5–2 mg/kg IV bolus

**Table 2:** Non-pharmacological strategies for analgosedation in the Neonatal Intensive Care Unit (NICU).

Category	Intervention	Description	Potential Benefits
Proximal strategies	Skin-to-skin contact (Kangaroo Care)	Infant placed directly on the parent’s chest, ensuring warmth and containment.	Diminishes pain response, supports physiologic stability, fosters bonding.
	Facilitated tucking	Limbs gently held in a flexed position close to the body.	Promotes self-soothing, reduces distress behaviors.
	Non-nutritive sucking	Pacifier or caregiver’s finger offered without feeding.	Provides calming input, decreases crying and grimacing.
	Breastfeeding	Feeding at the breast during or shortly after a painful procedure.	Combines nutritive, tactile, and emotional comfort.
	Oral sucrose/glucose	Small amount of sweet solution given before a noxious stimulus.	Activates endogenous analgesic mechanisms, shortens pain expression.
Distal strategies	Environmental adjustment	Regulation of light, sound, and odors in the NICU setting.	Creates a soothing atmosphere, limits overstimulation.
	Positioning and swaddling	Secure wrapping or supported positioning of the neonate.	Enhances containment, lowers procedural discomfort.
	Gentle touch/massage	Soft, rhythmic tactile stimulation.	Encourages relaxation, decreases physiologic stress signs.
Multisensory approaches	Music therapy	Exposure to calm, live or recorded music.	Improves stability, supports pain reduction and comfort.
	Combined interventions	Application of two or more strategies together (e.g., sucrose + facilitated tucking).	Produces additive analgesic and calming effects.

## Data Availability

All data are included in the manuscript.
